# PRP-loaded hydrogel for rotator cuff repair by promoting osteogenic differentiation and tendon regeneration

**DOI:** 10.3389/fbioe.2025.1655809

**Published:** 2025-10-30

**Authors:** Ru-Sen Zhang, Zhi-Yang Zheng, Guo-Qing Zhu, Chao-Rong Luo, Jing-Min Yuan, Yi-Lin Ye, Ze-Qin Zhuang, Ze-Zheng Liu, Qing-Chu Li, Xi-Zhao Huang

**Affiliations:** ^1^ Department of Pain Medicine, Guangdong Women and Children Hospital, Guangzhou, Guangdong, China; ^2^ Department of Orthopedics, The Third Affiliated Hospital of Southern Medical University, Guangzhou, Guangdong, China; ^3^ Department of Anesthesiology, Guangdong Women and Children Hospital, Guangzhou, Guangdong, China

**Keywords:** rotator cuff injury, platelet-rich plasma, biomaterials, hydrogel, tendon-bone interface

## Abstract

**Objective:**

Rotator cuff injuries are a common and challenging shoulder joint pathology that significantly impacts shoulder function and quality of life. Current treatments, particularly arthroscopic repair, face high failure rates due to the difficulty of regenerating the native tendon-bone interface. This study aimed to develop and evaluate a platelet-rich plasma–carboxymethyl chitosan–tannic acid composite hydrogel (PRP-CMCS-TA), designed to address these limitations and enhance the repair and regeneration of the tendon-bone interface.

**Methods:**

The composite hydrogel was synthesized by creating a CMCS-TA hydrogel via chemical cross-linking, which was then integrated with PRP. Its functionality was assessed through a series of *in vitro* biocompatibility, bioactivity, and sustained-release assays, and its efficacy in promoting tendon-bone interface regeneration was evaluated in a rat rotator cuff injury model using micro-CT, biomechanical testing, and histological analysis.

**Results:**

*In vitro* experiments demonstrated the biocompatibility and bioactivity of PRP-CMCS-TA. The hydrogel supported cell viability and proliferation, and sustained the release of PRP components, which are critical for promoting tenogenic and osteogenic differentiation. The *in vitro* results indicated that PRP-CMCS-TA could provide a suitable environment for cell growth and tissue repair. *In vivo* studies were conducted using a rat rotator cuff injury model. The model was divided into three groups: Control, CMCS-TA, and PRP-CMCS-TA. Micro-CT scans and histological analyses were performed at predetermined time points to evaluate the repair capability of the composite hydrogel. The results showed that PRP-CMCS-TA significantly promoted bone regeneration and achieved superior tendon-bone interface repair compared to the other groups.

**Conclusion:**

The PRP-CMCS-TA composite hydrogel exhibits good biocompatibility and effectively repairs the tendon-bone interface by promoting osteogenic differentiation and tendon regeneration. This study provides new insights into the application of bioactive materials for rotator cuff injuries and offers a promising strategy for improving the outcomes of rotator cuff repair. Further research and clinical trials are needed to translate these findings into practical applications and to establish the hydrogel as a standard treatment option for rotator cuff injuries.

## 1 Introduction

Rotator cuff tears are a major cause of shoulder pain and dysfunction, posing significant socioeconomic burdens (Zhao et al., 2022; Littlewood et al., 2018; Saccomanno et al., 2023). As populations age and physical activity increases, their incidence continues to rise (Weber and Chahal, 2020). Although arthroscopic repair and other surgical techniques have advanced, failure rates remain high (20%–94%) due to factors such as tear size, chronicity, and patient - related factors (Abtahi et al., 2015; Trasolini and Waterman, 2022; Burnier et al., 2019; Sripathi and Agrawal, 2024; Patel et al., 2016). The main challenge in rotator cuff repair is not only reattaching torn tendons to bone but also regenerating the native tendon - bone interface (TBI), a specialized tissue that dissipates mechanical stress and prevents stress concentration (Patel et al., 2016; Sripathi and Agrawal, 2024). The native TBI, or “enthesis,” has a complex structure transitioning from tendon to bone through fibrocartilage (Wang H. et al., 2024). However, after injury and surgical repair, the native enthesis fails to regenerate spontaneously, leading to the formation of disorganized fibrovascular scar tissue (Weeks et al., 2014). This tissue has poor biomechanical properties, deficient integration, and high susceptibility to re-tear (Uzun et al., 2021; Routledge et al., 2023). Biological limitations include hypovascularity at the repair site, insufficient recruitment and differentiation of progenitor cells, compromised extracellular-matrix synthesis, and an inability to re-establish the biochemical gradients and structural complexity required for functional healing (Hegedus et al., 2010; Nho et al., 2008; Yang et al., 2023).

Current strategies to enhance rotator cuff repair involve biological adjuvants and tissue engineering (Zou et al., 2023; Saveh-Shemshaki et al., 2019). Platelet rich plasma (PRP) has garnered attention due to its high - concentration autologous growth factors, which modulate inflammation, stimulate angiogenesis, drive cell proliferation, and boost ECM synthesis (Collins et al., 2021; Le et al., 2018). However, its clinical efficacy is still debated. Key limitations include the rapid clearance of bioactive factors from the injury site and their short half-life after direct injection or application, undermining PRP’s regenerative potential (Khandan-Nasab et al., 2025). Additionally, liquid PRP lacks structural support, falling short of the mechanical demands of the healing attachment site (Qi et al., 2024).

To overcome PRP’s limitations and provide three-dimensional structural guidance, hydrogel-based scaffolds have emerged as a promising platform for tendon–bone interface (TBI) regeneration. Hydrogels can mimic the natural ECM, offer temporary mechanical support, deliver bioactive molecules in a sustained way, and serve as a matrix for cell infiltration and tissue growth (Bakadia et al., 2023; Yun et al., 2025). Chitosan and its derivatives have excellent biocompatibility, biodegradability, antibacterial properties, and cell adhesion - promoting ability (Zhang X. et al., 2024; Youssef et al., 2015). Carboxymethyl chitosan (CMCS) further augments these qualities, enhancing water solubility and supplying versatile functional groups for subsequent chemical modification. Tannic acid (TA) has gained interest in biomaterials due to its superior adhesion, antioxidant activity, metal-ion chelation ability, and capacity to form strong physical cross - links with polymers like chitosan (Guo et al., 2022; Zhang Y. et al., 2024; Xu et al., 2024). TA can also bind and stabilize growth factors in PRP, potentially mitigating rapid diffusion and degradation (Niu et al., 2024).

Integrating PRP into a functional hydrogel matrix presents a compelling strategy that combines PRP’s biological cues with the scaffold’s structural and sustained - release benefits. The CMCS - TA hydrogel system offers a suitable platform, with CMCS providing biocompatibility and structural integrity, and TA enabling cross-linking, conferring bioadhesion and protective properties, and creating a network for controlled PRP-derived factor release. This combined approach addresses challenges such as rapid PRP clearance, lack of mechanical support, and the need for a biomimetic environment conducive to TBI regeneration.

Based on this rationale, we hypothesize that a PRP-loaded CMCS–TA composite hydrogel (PRP–CMCS–TA) will create an optimal regenerative microenvironment for TBI healing after rotator cuff repair. This hydrogel is expected to combine the biocompatibility and structural stability of CMCS-TA with the sustained release of bioactive factors from PRP, thereby enhancing the cellular responses crucial for regeneration of the attachment. This study aims to synthesize and characterize the novel PRP-CMCS-TA composite hydrogel, evaluate its in - vitro biocompatibility and bioactivity, assess its effects on relevant cell types, its ability to sustain PRP release, and its capacity to induce local targeted osteogenic and tendinous differentiation of cells. Finally, using comprehensive micro-CT and histological analysis, its efficacy in promoting functional TBI regeneration will be determined in a mature rat model of rotator cuff injury. We anticipate that PRP–CMCS–TA will outperform both untreated controls and CMCS–TA alone, thereby offering a clinically translatable, bioactive scaffold strategy for enhanced rotator cuff repair.

## 2 Experimental section

### 2.1 Materials

Carboxymethyl chitosan (CMCS, Mw = 543.51), Tannic acid (TA, Mw = 1701.2 Da), sodium bicarbonate (NaHCO_3_, Mw = 84.01) were purchased from Sigma-Aldrich. All chemicals were used without further purification.

### 2.2 Preparation of CMCS-TA

The CMCS-TA hydrogel was prepared according to the following protocol. First, three solutions were prepared: 5% (w/v) carboxymethyl chitosan (CMCS) solution, 0.2 g/mL tannic acid (TA) solution, and 4% (w/v) sodium bicarbonate (NaHCO_3_) solution. Briefly, 2 mL of TA solution was mixed with 1 mL of NaHCO_3_ solution in a centrifuge tube and allowed to stand for 1 min. Subsequently, 2.5 mL of 5% (w/v) CMCS solution was added to the mixture and thoroughly vortexed. The CMCS-TA hydrogel formed within 1 min after homogenization.

A portion of the freshly prepared CMCS-TA hydrogel was lyophilized using a freeze dryer for subsequent characterization. The remaining hydrogel was reserved for further preparation of the composite hydrogel PRP-CMCS-TA by incorporating platelet-rich plasma (PRP).

To obtain scanning electron microscopy images of CMCS-TA, a TESCAN microscope (Czech) was utilized. Fourier transform infrared spectra (FTIR) were measured using a Thermo Scientific FTIR spectrometer. X-ray diffraction (XRD) measurements of CMAS, TA and CMCS-TA were conducted using a Bruker X-ray powder diffractometer.

### 2.3 PRP isolation and PRP-CMCS-TA composite hydrogel preparation

Platelet-rich plasma (PRP) was isolated from whole blood collected via cardiac puncture from adult, male Sprague-Dawley rats (8–10 weeks old, n = 6), which were separate from the animals used in the surgical rotator cuff injury model. Whole blood was collected from rat hearts using EDTA-anticoagulated centrifuge tubes. Platelet-rich plasma (PRP) was isolated through differential centrifugation. Briefly, the whole blood was first centrifuged at 300 *g* for 15 min at room temperature to separate erythrocytes. The supernatant (plasma containing platelets and leukocytes) was then collected and subjected to a second centrifugation at 700 *g* for 10 min to pellet the platelets. The upper plasma layer was partially discarded, and the platelet pellet was gently resuspended in the remaining plasma to obtain concentrated PRP.

The PRP-CMCS-TA composite hydrogel was prepared by blending the CMCS-TA hydrogel with PRP at a 5:1 (v/v) ratio under gentle stirring to ensure homogeneous dispersion of PRP within the hydrogel matrix. Subsequently, calcium chloride (CaCl_2_) was added at a 10:1 (v/v) ratio to PRP. The resulting PRP-CMCS-TA hydrogel was immediately used for subsequent experiments.

### 2.4 Characterization of PRP-CMCS-TA

To obtain scanning electron microscopy images of PRP-CMCS-TA, a TESCAN microscope (Czech) was utilized. To assess the release kinetics of growth factors (VEGF、TGF-β and PDGF) from PRP-CMCS-TA hydrogels, sterile PRP-CMCS-TA and PRP samples were placed in 24-well plates. α-MEM medium was added to each well, followed by incubation in a humidified incubator at 37 °C with 5% CO_2_ for 14 days. The culture medium was collected and replaced with fresh medium every 48 h. Supernatants collected at days 1, 3, 5, and 7 were analyzed using ELISA to quantify the concentrations of VEGF, TGF-β and PDGF for release profile characterization. The release kinetics experiment was performed with n = 4 independent samples per group, and ELISA measurements for each sample were conducted in duplicate.

### 2.5 Isolation and culture of bone marrow mesenchymal stem cells (BMSCs)

Rat bone marrow mesenchymal stem cells (BMSCs) were isolated from the femurs and tibias of 8 male Sprague-Dawley rats (4-week-old) following an established protocol (Ni et al., 2025). These primary cells were then expanded *in vitro* through passages 3-5 to obtain sufficient cell numbers for all subsequent *in vitro* experiments. Briefly, bone marrow was flushed from the femurs and tibias using complete culture medium (α-MEM supplemented with 10% FBS and 1% penicillin/streptomycin). The collected cells were cultured at 37 °C in a 5% CO_2_ humidified atmosphere. Non-adherent cells were removed during medium changes every 3 days. BMSCs at passages 3-5 were used for all experiments.

### 2.6 Culture of tendon-derived stem cells (TDSCs)

Rat tendon-derived stem cells (TDSCs) were isolated from the Achilles tendons of 8 male Sprague-Dawley rats (4-week-old) following an established protocol (Yu et al., 2024). These primary cells were then expanded *in vitro* through passages 3-5 to obtain sufficient cell numbers for all subsequent *in vitro* experiments. The tendons were minced and digested in collagenase type I solution for 4 h at 37 °C. After filtration and centrifugation, the obtained cells were cultured in growth medium (α-MEM containing 10% FBS, 1% penicillin/streptomycin, and 2 ng/mL bFGF). TDSCs at passages 3-5 were utilized for the *in vitro* experiments.

### 2.7 Biocompatibility of PRP-CMCS-TA

Bone marrow mesenchymal stem cells (BMSCs) were utilized to evaluate the cytotoxicity of PRP-CMCS-TA hydrogels. To obtain extracts for biocompatibility testing, PRP-CMCS-TA and CMCS-TA hydrogels were prepared under sterile conditions and immersed in complete α-MEM medium at a ratio of 200 mg of hydrogel per 10 mL of medium. The mixture was incubated at 37 °C for 24 h. Subsequently, the mixture was centrifuged, and the supernatant (the extract) was collected and used for the CCK-8 assay. PBS containing α-MEM medium serving as the control. BMSCs were cultured in 96-well plates at a density of 5,000 cells per well. Cell proliferation after 24 h and 48 h of co-culture with extracts or PBS was assessed using the CCK-8 assay. Absorbance at 450 nm (OD) was measured using a microplate reader. Each group contained at least six replicates, and results were averaged. Furthermore, to more accurately assess cytocompatibility, live-cell staining kits were employed to stain BMSCs. Stained cells were observed under an inverted fluorescence microscope (Leica, Germany), with quantitative analysis performed using ImageJ software.

### 2.8 Cell recruitment *in vitro*

Transwell migration assays were performed using permeable inserts (Corning, United States) placed in 24-well plates containing α-MEM. BMSCs were seeded in the upper chambers containing different experimental treatments and incubated for 24 h. Cells remaining on the upper surface were removed using cotton swabs. Subsequently, cells were fixed with 5% glutaraldehyde solution for 30 min and stained with crystal violet for 15 min. Migrated cells were observed and imaged under an Olympus microscope.

### 2.9 Verification of PRP-CMCS-TA regulating osteogenic differentiation of BMSCs

BMSCs were seeded in 24-well plates and cultured in complete osteogenic medium supplemented with different experimental treatments. The medium was refreshed every 48 h. For ALP staining, cells were fixed with 4% paraformaldehyde (PFA) after 7 and 14 days of osteogenic induction, followed by staining using a BCIP/NBT Alkaline Phosphatase Color Development Kit. For Alizarin Red S staining, cells were fixed with 4% PFA after 21 days of osteogenic culture and stained with Alizarin Red S solution. Stained cells were visualized under an inverted fluorescence microscope and quantified using ImageJ software.

Additionally, immunofluorescence staining was performed to confirm the capacity of PRP-CMCS-TA to promote osteogenic differentiation. Following 5 days of culture under the protocol, BMSCs from different groups were subjected to immunofluorescence staining using antibodies against ALP and Runx2. Immunofluorescent cells were then observed and imaged under an inverted fluorescence microscope.

### 2.10 Validation of PRP-CMCS-TA regulation of TDSCs tendon differentiation

TDSCs were seeded in complete α-MEM medium supplemented with different experimental treatments and cultured for 5 days. Cells were then fixed with 4% paraformaldehyde (PFA). Immunofluorescence staining was performed on TDSCs from each group using antibodies against Col I.-Stained cells were observed and imaged under an inverted fluorescence microscope. Semi-quantitative analysis was conducted using ImageJ software.

### 2.11 Animal study design and surgical procedure

All animal care and experimental procedures were approved by the Institute of Biological and Medical Engineering, Guangdong Academy of Sciences (Approval No. K2024-01-157-479). A total of twenty-four 8-10-week-old male Sprague-Dawley rats were randomly divided into three groups (n = 8 per group): Control group, CMCS-TA group, and PRP-CMCS-TA group. The sample size (n = 8 per group) was determined based on previous literature utilizing similar rat rotator cuff repair models, where this group size was sufficient to demonstrate significant effects.

A rat rotator cuff injury model was established as previously described (Wang Y. et al., 2024). Briefly, rats were anesthetized via intraperitoneal injection of a mixture of ketamine (100 mg/kg) and xylazine (10 mg/kg). Following the onset of deep anesthesia, a longitudinal incision was made over the anterolateral shoulder to expose the supraspinatus tendon. The deltoid muscle was incised to enhance visualization. The tendon was then sharply detached from its insertion site at the greater tuberosity of the humerus. Bone tunnels were created using a curved needle. After debridement of residual tissue at the supraspinatus tendon footprint, a total volume of 20 μL of the respective hydrogel was injected at the tendon-bone interface (footprint region and freshened surface) using a micro-syringe. In the control group, no hydrogel was injected, and the retracted tendon was reduced through the bone trough. The supraspinatus tendon was subsequently sutured back to the greater tuberosity using non-absorbable sutures. Finally, the joint capsule and skin were meticulously closed in layers. Unilateral surgery (on the right shoulder) was performed on all animals.

For postoperative analgesia, buprenorphine (0.05 mg/kg) was administered subcutaneously every 12 h for 48 h. To prevent infection, penicillin (40,000 U/day) was administered intramuscularly for 3 days following surgery. All animals were monitored daily for signs of distress or complications and were allowed free activity in their cages postoperatively.

### 2.12 Micro-CT analysis

At 8 weeks postoperatively, Sprague-Dawley rats were euthanized, and shoulder joint tissues were harvested. Four specimens per group underwent micro-computed tomography (micro-CT) analysis. Three-dimensional reconstructions were generated from the acquired data using Mimics software (version 20.0). Bone mineral density (BMD), bone volume fraction (BV/TV), and separation degree of bone trabeculae (Tb.sp) were quantified using LaTheta software. All specimens underwent initial pre-scanning followed by high-resolution scanning of the region of interest (ROI).

### 2.13 Biomechanical testing

At 8 weeks post-rotator cuff repair (RCR) surgery, shoulder joints were harvested from four rats per group for analysis. Surrounding soft tissues and ligaments were meticulously dissected to isolate the reconstructed supraspinatus tendon-humerus complex. Specimens were tested using a universal electronic testing machine. Each sample was wrapped in saline-soaked gauze to prevent dehydration and minimize mechanical interference. After preconditioning with 0.1 N preload, specimens were subjected to uniaxial tensile loading at an elongation rate of 10 mm/min until failure. Failure load was recorded.

### 2.14 Histological analysis

At 8 weeks postoperatively, rats were euthanized, and supraspinatus tendon-humerus complexes were harvested. Specimens were fixed in 4% paraformaldehyde, followed by decalcification in 0.5 M EDTA at 37 °C for 4 weeks. After dehydration and paraffin embedding, samples were sectioned coronally at 4 μm thickness. Sections were stained with hematoxylin and eosin (H&E) and toluidine blue (TB). Stained slides were examined under an optical microscope (Olympus, Japan). A semi-quantitative histologic scoring system was employed to evaluate the tendon-bone interface. Scoring criteria included: Angiogenesis (1 = abundant, 4 = sparse), Cellularity (1 = hypercellular, 4 = hypocellular), Tissue continuity (1 = discontinuous transition <25%, 4 = continuous transition >75%), Fibrocartilage formation (1 = <25%, 4 = >75%), Tidemark integrity (1 = coverage <25%, 4 = coverage >75%). Higher total scores indicate superior tendon-bone healing. Metachromatic area in TB-stained slides was quantified using ImageJ software. Histological assessments were performed by two independent observers blinded to group allocation.

### 2.15 Statistical analysis

Statistical analyses were performed using SPSS software (version 26.0, United States), and results were expressed as mean ± standard deviation. Graphs for data visualization were generated using GraphPad Prism software (version 9, SUSA). Quantitative analysis among multiple groups was performed using one-way ANOVA followed by Tukey’s Post-Hoc Test. Pairwise comparisons were conducted using Student’s t-test. A value of *p* < 0.05 was considered a statistically significant difference. *, ** and *** represent *p* < 0.05, *p* < 0.01 and *p* < 0.001, respectively.

## 3 Result and discussion

### 3.1 Synthesis and characterizations of CMCS-TA

**FIGURE 1:**
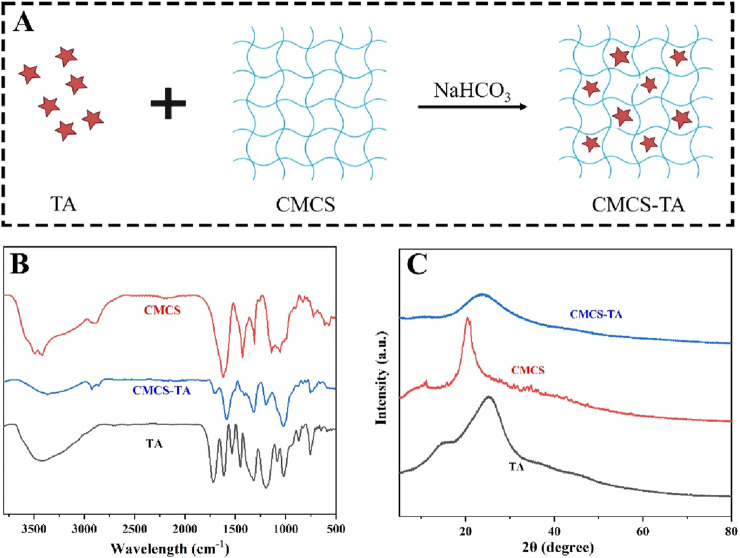
**(A)** Schematic illustration of CMCS-TA hydrogel synthesis. **(B)** FTIR spectra of TA, CMCS, and CMCS-TA. **(C)** XRD patterns of TA, CMCS, and CMCS-TA.

As shown in Figure 1A, CMCS and TA are cross - linked via hydrogen bonds in a sodium bicarbonate environment to form the CMCS - TA hydrogel. Successful cross - linking of TA and CMCS is evidenced by the FTIR spectra of CMCS, TA, and CMCS - TA (Figure 1B). In Figure 1B, the O- H stretching vibration peak of phenolic hydroxyl groups in CMCS - TA is observed. Moreover, the characteristic absorption peaks of CMCS and TA undergo shifts and show increased intensity in the ranges of 1,600–1,650 cm^-1^ and 3,200–3,600 cm^-1^ due to enhanced hydrogen bond interactions (Zhou et al., 2023). XRD results of CMCS, TA, and CMCS - TA further confirm the successful synthesis of CMCS - TA. The XRD pattern of CMCS exhibits a broad halo peak in the low - angle region (e.g., 10°–30°), and TA also displays an amorphous XRD pattern (Huang et al., 2024). After CMCS and TA are combined to form CMCS - TA, the XRD pattern reveals lower crystallinity than that of pure CMCS and TA. This is attributed to the interactions between CMCS and TA disrupting their original crystalline structures, thereby reducing the overall crystallinity of the composite material.

### 3.2 Synthesis and characterizations of PRP-CMCS-TA

**FIGURE 2:**
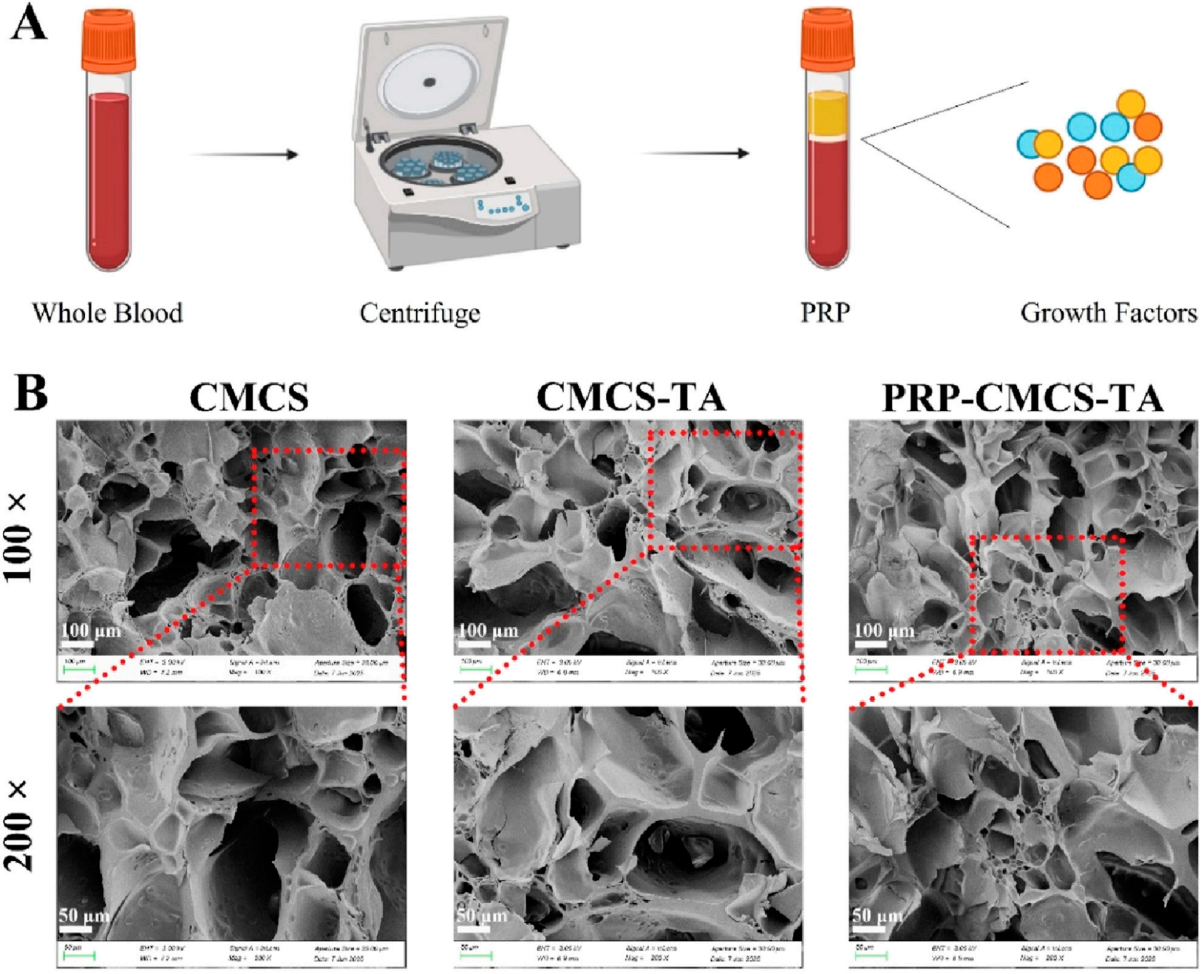
**(A)** Schematic diagram of PRP acquisition. **(B)** SEM images of CMCS, CMCS-TA, and PRP-CMCS-TA hydrogels.

Figure 2A shows the process of obtaining PRP. The SEM images show that the CMCS hydrogel has a higher porosity with relatively large and irregular pores. In contrast, the CMCS - TA and PRP-CMCS-TA hydrogels display a more compact structure with smaller and more uniform pores. This change in pore structure is likely due to the cross - linking effect of TA, which alters the arrangement of CMCS chains and reduces the space between them, thereby decreasing porosity. The similar porosities of the PRP-CMCS-TA and CMCS-TA scaffolds suggest that the addition of PRP does not substantially affect the pore structure formed by the CMCS - TA network.

**FIGURE 3:**
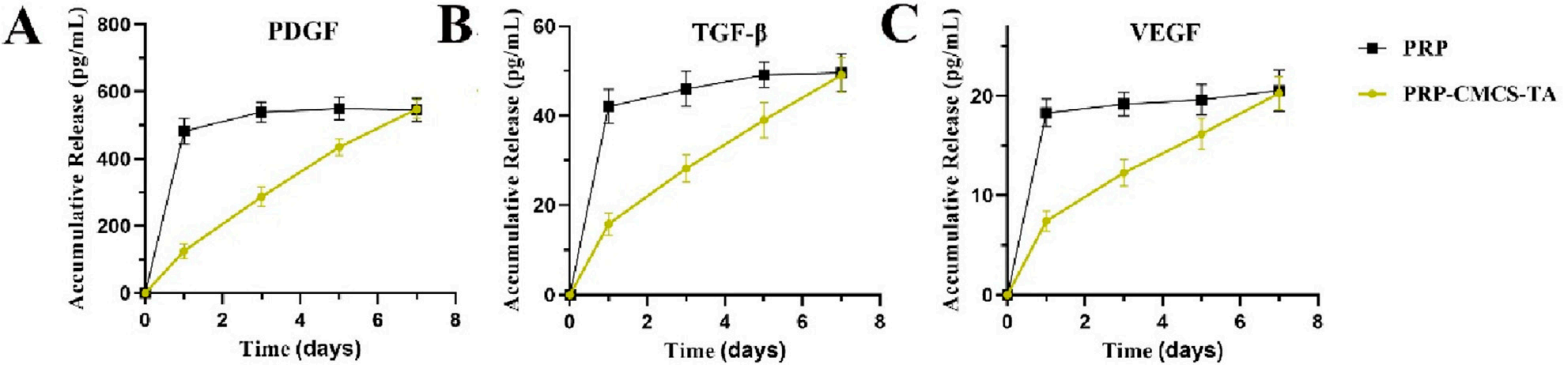
Release curves of PDGF **(A)**, TGF-β **(B)**, and VEGF **(C)** from PRP-CMCS-TA hydrogel determined by ELISA kits.

As shown in Figure 3, the release profiles of PDGF, TGF-β, and VEGF from the PRP-CMCS-TA hydrogel were characterized and compared to those from PRP alone. While PRP alone exhibited a characteristic burst release, with over 80% of each growth factor released within the first 24–48 h, the PRP-CMCS-TA hydrogel demonstrated a markedly more sustained and controlled release pattern over the 7-day period.

For the PRP-CMCS-TA group, all three growth factors showed an initial rapid release phase within the first few days, likely due to the diffusion of surface-associated and loosely bound molecules from the hydrogel matrix. This was followed by a gradual and prolonged release phase, indicating the successful incorporation and retention of growth factors within the hydrogel network. Specifically, the cumulative release of PDGF (Figure 3A) reached approximately 400 pg/mL in the initial phase and steadily increased to around 550 pg/mL by day 7. Similarly, TGF-β (Figure 3B) increased to about 37 pg/mL by day 5, then rose gradually to 43 pg/mL by day 7. VEGF (Figure 3C) showed the lowest release level, accumulating to around 20 pg/mL by day 7.

In contrast, PRP alone released most of its growth factors rapidly, resulting in a high initial concentration that quickly plateaued, highlighting its limitations as a sustained delivery system. These results confirm that the PRP-CMCS-TA hydrogel effectively mitigates the burst release phenomenon associated with PRP alone and provides a sustained release of bioactive factors, which is advantageous for long-term tissue regeneration processes.

### 3.3 Verify the cell viability and migration of PRP-CMCS-TA *in vitro*

**FIGURE 4:**
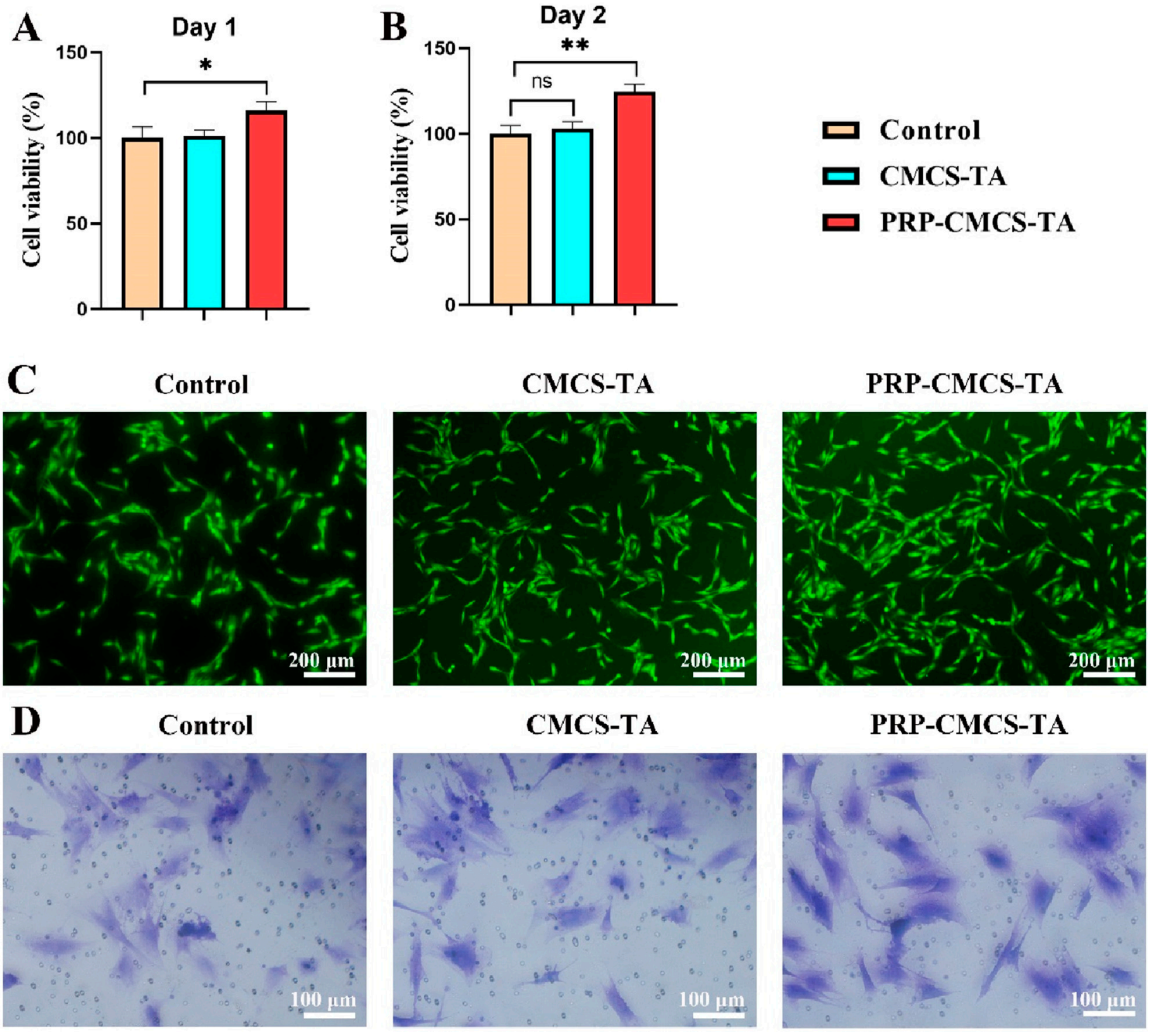
Evaluation of the cytocompatibility of PRP-CMCS-TA hydrogel. **(A,B)** CCK-8 assay for cytotoxicity on day 1 and day 2. Data are presented as mean ± SD (n = 6 independent replicates). **(C)** Live cell staining of BMSCs in three groups. **(D)** Images of Transwell assays for BMSCs in three groups. Statistical significance was determined by one-way ANOVA. *p < 0.05, **p < 0.01,”ns” represents no significant difference.

Establishing the biocompatibility of PRP-CMCS-TA is essential before *in vitro* and *in vivo* applications to ensure it supports cell viability and function. Figure 4 presents the evaluation of the cytocompatibility of PRP-CMCS-TA hydrogel. Figures 4A,B display the results of the CCK-8 assay for cytotoxicity on day 1 and day 2. On day 1, the cell viability of the PRP-CMCS-TA group was significantly higher (p < 0.05) than that of the control and CMCS-TA groups. On day 2, the PRP-CMCS-TA group showed a notable increase in cell viability compared to the control group, indicated by **p < 0.01, while the difference between the CMCS-TA and control groups was not significant. These data indicate that the PRP–CMCS–TA hydrogel possesses excellent cytocompatibility and may actively promote cell proliferation over time. The PRP-CMCS-TA group displays more green fluorescence, indicating higher cell activity and proliferation. Transwell migration assays (Figure 4D) further show a higher number of BMSCs migrating to the lower chamber in the PRP–CMCS–TA group, underscoring enhanced cell motility. Overall, the PRP–CMCS–TA hydrogel demonstrates favorable cytocompatibility, effectively supporting both proliferation and migration of BMSCs.

### 3.4 *In vitro* osteogenic differentiation performance

**FIGURE 5:**
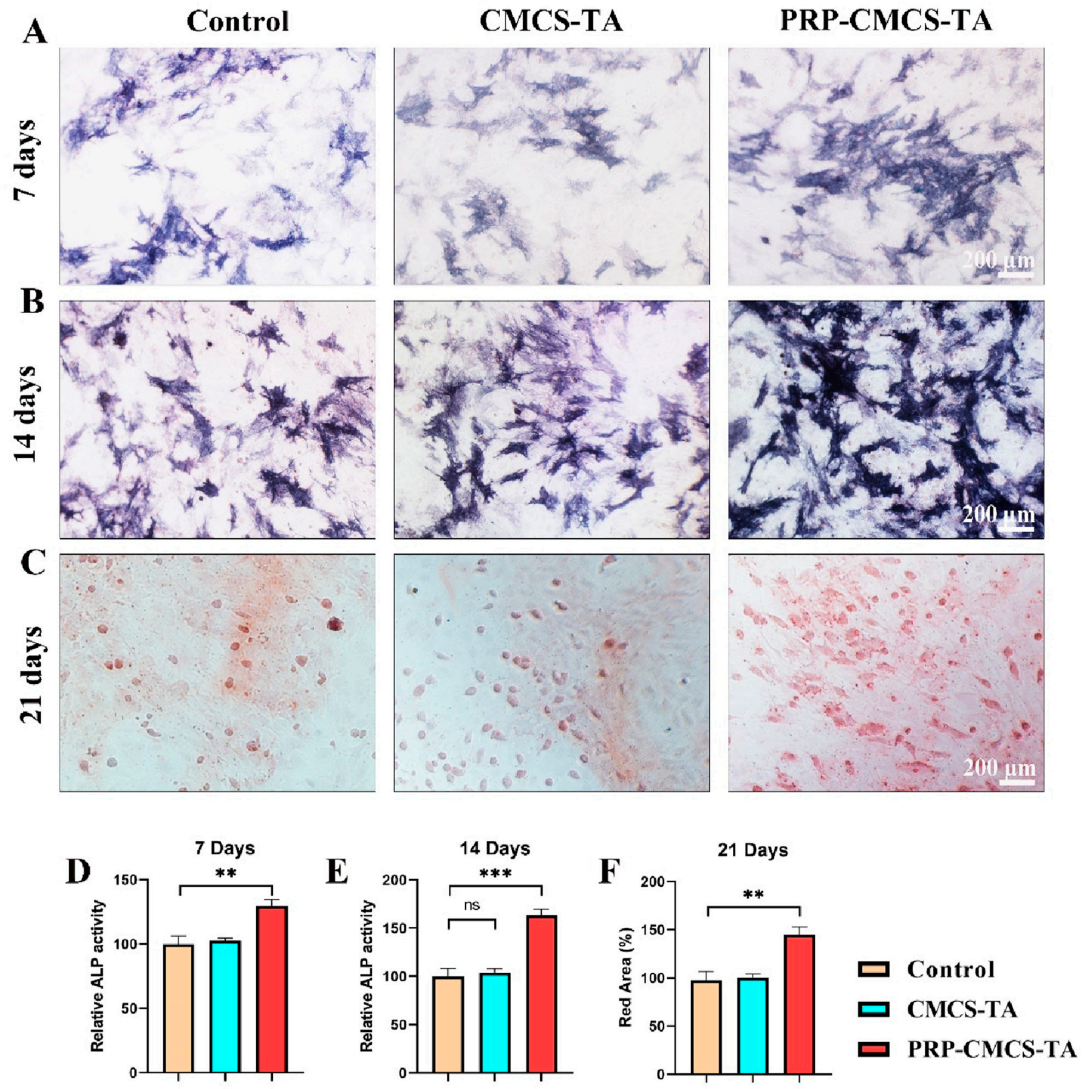
Assessment of the osteogenic differentiation - promoting ability of PRP-CMCS-TA hydrogel. **(A,B)** ALP staining images of BMSCs after 7 and 14 days of different interventions. **(C)** Alizarin Red S staining images of BMSCs after 21 days of different interventions. **(D,F)** Semi-quantitative analysis of ALP activity (**(D)** day 7, **(E)** day 14) and mineralized nodule formation (**(F)** day 21) shown in **(A–C)**. Data are presented as mean ± SD (n = 6 independent biological samples per group). Statistical analysis was performed using one-way ANOVA. **p < 0.01, ***p < 0.001,”ns” represents no significant difference.

Osteogenic differentiation is critical for bone regeneration at the tendon - bone interface, so assessing the PRP-CMCS-TA hydrogel’s ability to induce osteogenesis in BMSCs is vital for its potential application in rotator cuff repair (Luzzi et al., 2023; Ni et al., 2025). Figure 5 demonstrates the capacity of PRP-CMCS-TA hydrogel to promote osteogenic differentiation. Figures 5A,B show ALP staining images of BMSCs after 7 and 14 days of various interventions. On day 7, the PRP-CMCS-TA group has more intense blue staining than the other groups, suggesting higher ALP activity, a marker of early osteogenic differentiation. This difference is even more pronounced on day 14. Figure 5C presents Alizarin Red S staining images of BMSCs after 21 days. The PRP-CMCS-TA group displays more red mineralized nodules, indicating advanced osteogenic differentiation and mineralization. Figure 5D-F display the semi-quantitative analysis of ALP staining on days 7 and 14, and Alizarin Red S staining on day 21. On day 7, the PRP-CMCS-TA group has significantly higher ALP activity than the control group. On day 14, the PRP-CMCS-TA group still shows higher ALP activity than the control group. On day 21, the PRP-CMCS-TA group has a markedly larger red area than the control group. Overall, the PRP-CMCS-TA hydrogel effectively enhances osteogenic differentiation of BMSCs at different stages.

**FIGURE 6:**
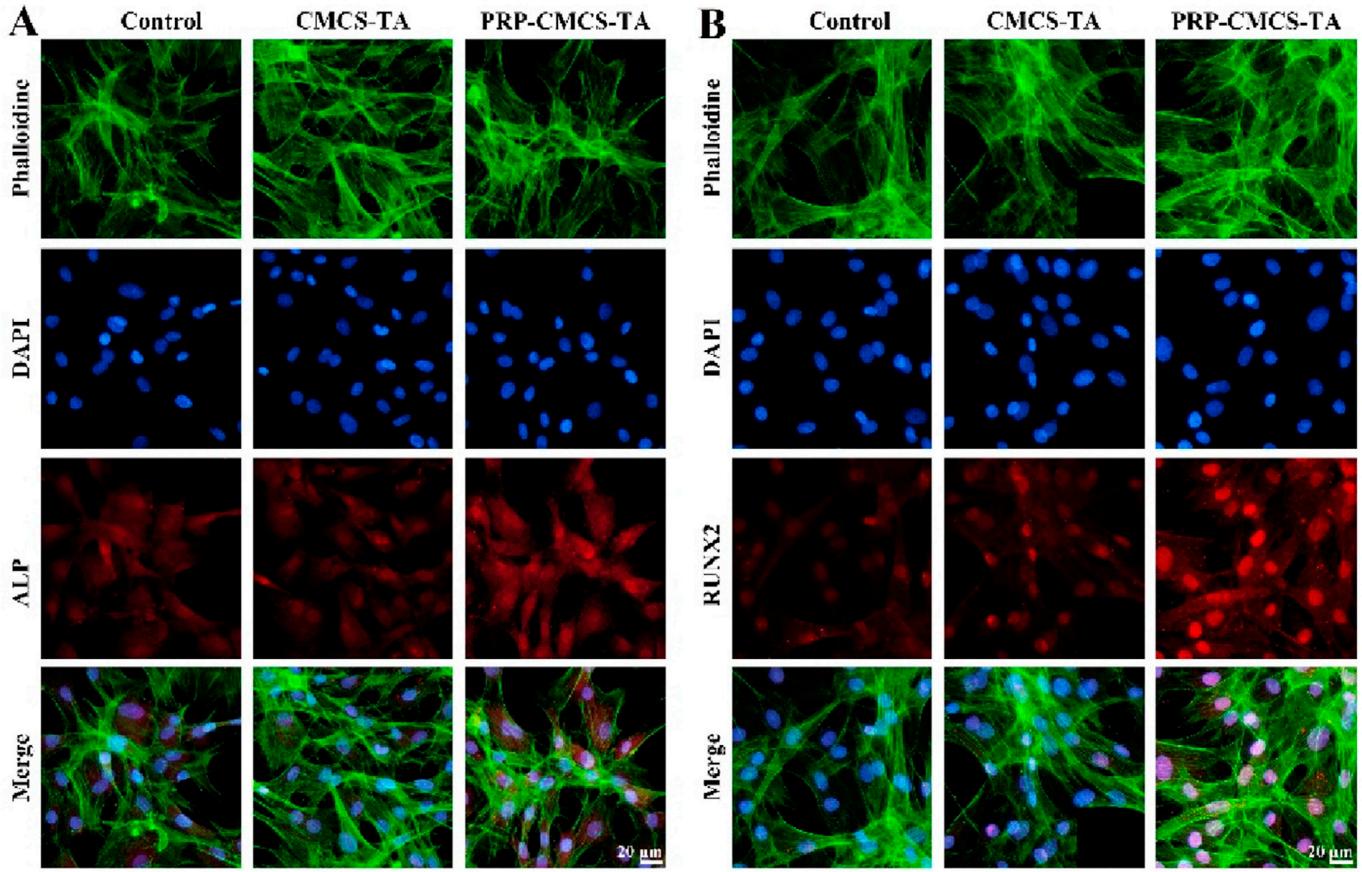
Evaluation of the osteogenic differentiation - promoting ability of PRP-CMCS-TA hydrogel via immunofluorescence staining. **(A)** ALP immunofluorescence staining images of cells in three groups obtained by fluorescence microscope. **(B)** RUNX2 immunofluorescence staining images of cells in three groups obtained by fluorescence microscope.

Figure 6 evaluates the osteogenic differentiation - promoting ability of PRP-CMCS-TA hydrogel via immunofluorescence staining. Figure 6A shows ALP immunofluorescence staining images of cells in three groups. The PRP-CMCS-TA group displays stronger red fluorescence than the other groups, indicating higher ALP expression. Immunofluorescence assays (Figure 6) further substantiate these results. ALP and RUNX2 signals (red fluorescence) are substantially stronger in the PRP–CMCS–TA group than in the other groups (Figures 6A,B), and merged images confirm nuclear and cytoskeletal co-localization. Thus, the PRP–CMCS–TA hydrogel robustly upregulates key osteogenic markers, underscoring its superior capacity to drive BMSC osteogenesis.

### 3.5 *In vitro* tendon regeneration performance

**FIGURE 7:**
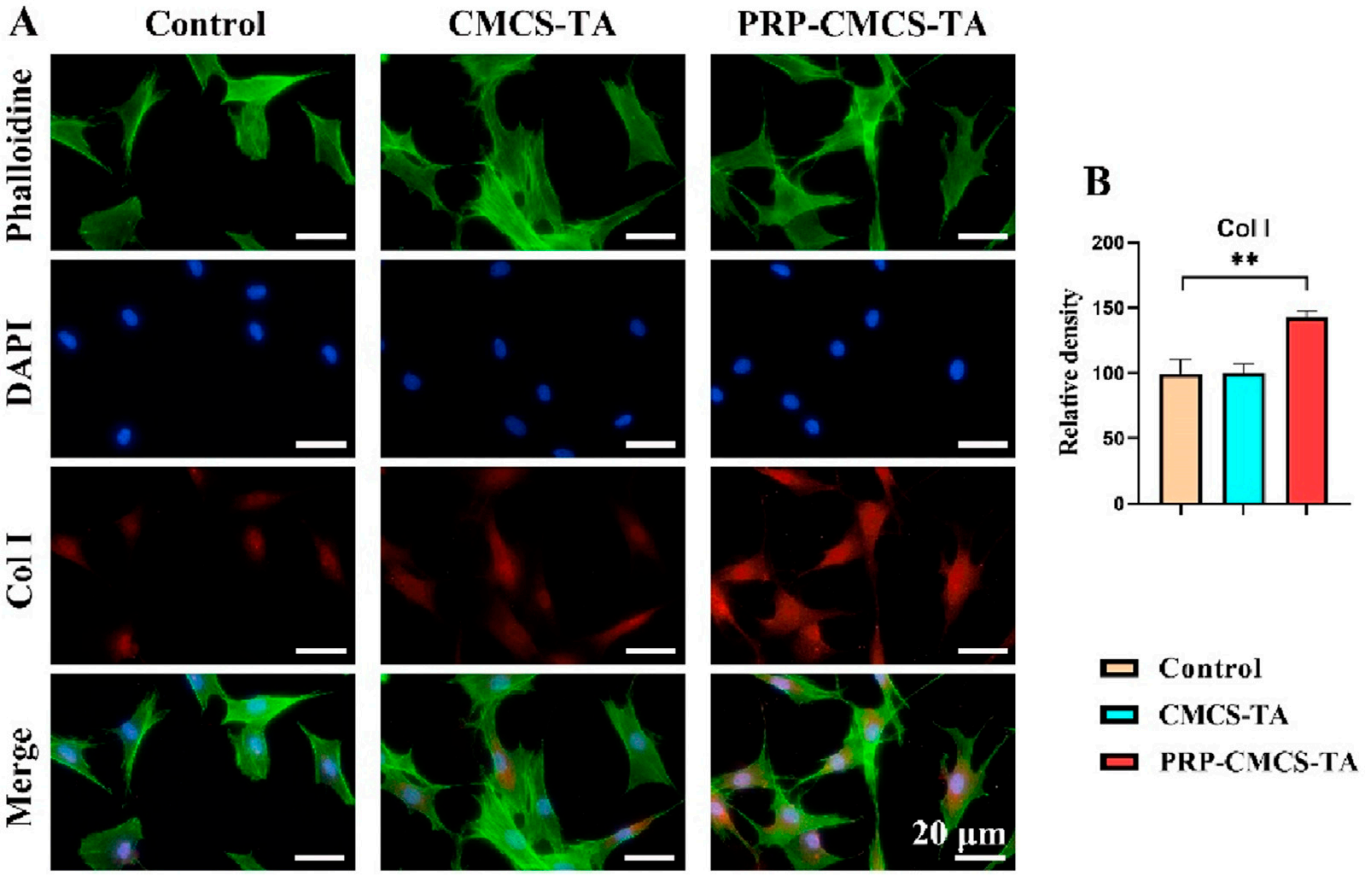
Assessment of the tendon regeneration - promoting ability of PRP-CMCS-TA hydrogel via immunofluorescence staining. **(A)** Col I immunofluorescence staining images of TDSCs in three groups obtained by fluorescence microscope. **(B)** Semi-quantitative analysis of Col I immunofluorescence intensity shown in **(A)**. Data are presented as mean ± SD (n = 6 independent replicates). Statistical significance was determined by one-way ANOVA. **p < 0.01.

Confirming the hydrogel’s ability to promote tendon regeneration is crucial for its application in rotator cuff repair, as this directly impacts the healing of the tendon part of the interface (Yu et al., 2024; Yea et al., 2020). Figure 7 presents the assessment of the tendon regeneration-promoting ability of PRP-CMCS-TA hydrogel. Figure 7A shows the Col I immunofluorescence staining images of TDSCs in three groups. The PRP-CMCS-TA group shows more intense red fluorescence, suggesting higher Col I expression. Figure 7B presents the semi - quantitative analysis of Col I. The PRP-CMCS-TA group has a significantly higher relative density than the control group. Overall, the PRP-CMCS-TA hydrogel effectively promotes Col I expression in TDSCs, indicating its potential to enhance tendon regeneration.

### 3.6 *In vivo* study of rat rotator cuff tears (RCT) model

After the establishment of the rat rotator cuff injury model, we divided the rats into three groups: the control group, the CMCS-TA group, and the PRP-CMCS-TA group. We evaluated the efficacy of the hydrogel in repairing rotator cuff injuries through micro-CT, mechanical analysis, and histological analysis.

### 3.7 Micro-CT analysis and biomechanical testing

**FIGURE 8:**
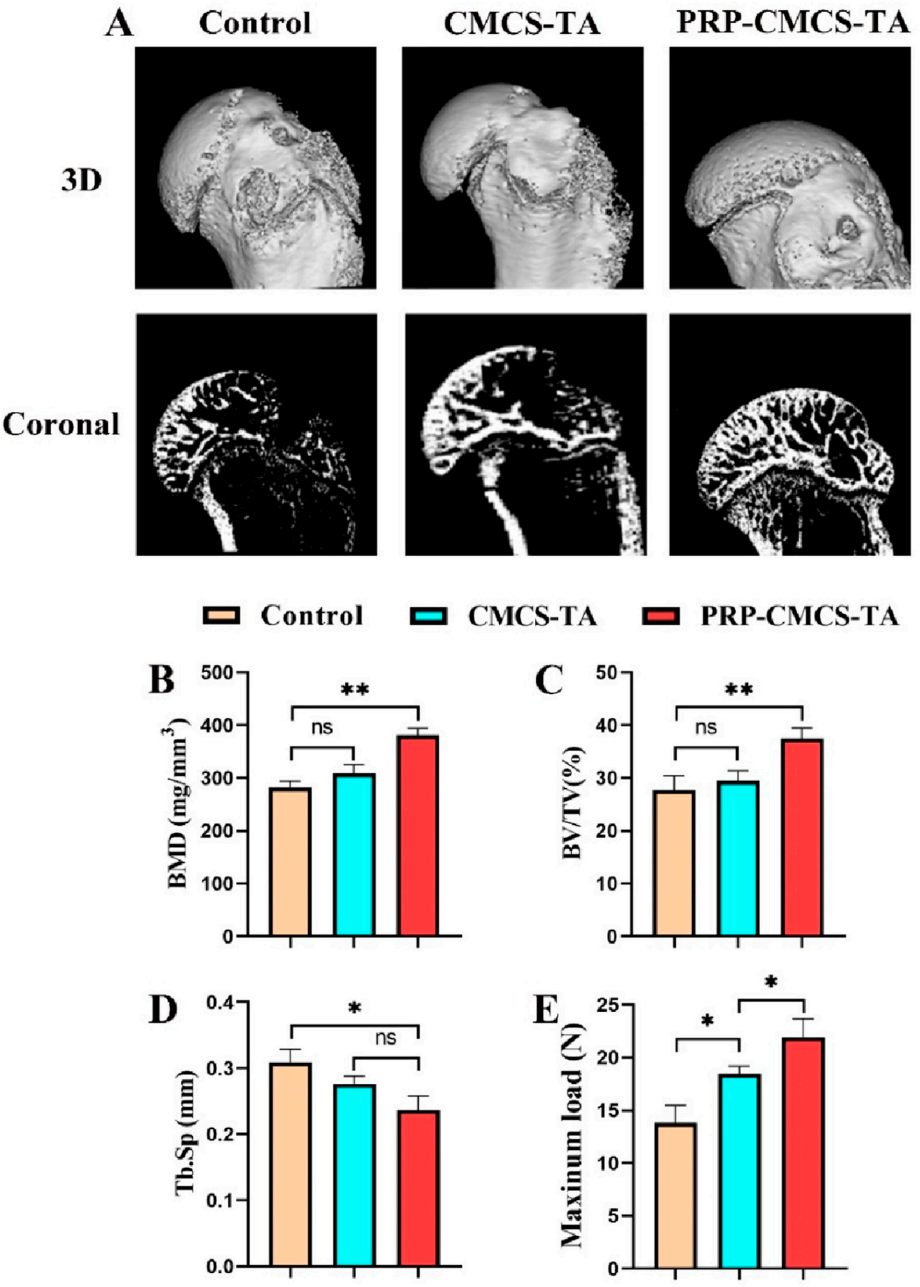
Micro - CT scanning and mechanical property testing to evaluate the effects of PRP-CMCS-TA. **(A)** 3D reconstruction images and coronal images of micro - CT of rat proximal humerus at 8 weeks post - operation. Quantitative analysis of micro-CT parameters: **(B)** Bone Mineral Density (BMD), **(C)** Bone Volume/Total Volume (BV/TV), and **(D)** Trabecular Separation (Tb.Sp). **(E)** Results of biomechanical testing showing the ultimate failure load of the repaired tendon-bone complex. Data are presented as mean ± SD (n = 4 independent animals per group for micro-CT; n = 4 independent specimens per group for biomechanical testing). Statistical analysis was performed using one-way ANOVA. *p < 0.05, **p < 0.01*,*”ns” represents no significant difference.

Figure 8 presents the results of micro - CT scanning and mechanical property testing to evaluate the effects of PRP-CMCS-TA. Figure 8A shows the 3D reconstruction and coronal images of micro - CT for rat proximal humerus 8 weeks post - operation. The PRP-CMCS-TA group demonstrates more complete bone regeneration and better integration with the surrounding tissue. The PRP-CMCS-TA group has significantly higher BMD and BV/TV than the control group. In terms of Tb. Sp, the PRP-CMCS-TA group has a lower value than the control group. Figure 8E shows the mechanical testing data. The PRP-CMCS-TA group has a higher maximum load than the control group. Overall, these results demonstrate that PRP-CMCS-TA hydrogel effectively promotes bone regeneration and enhances the mechanical properties of the tendon - bone interface.

### 3.8 Histological analysis

**FIGURE 9:**
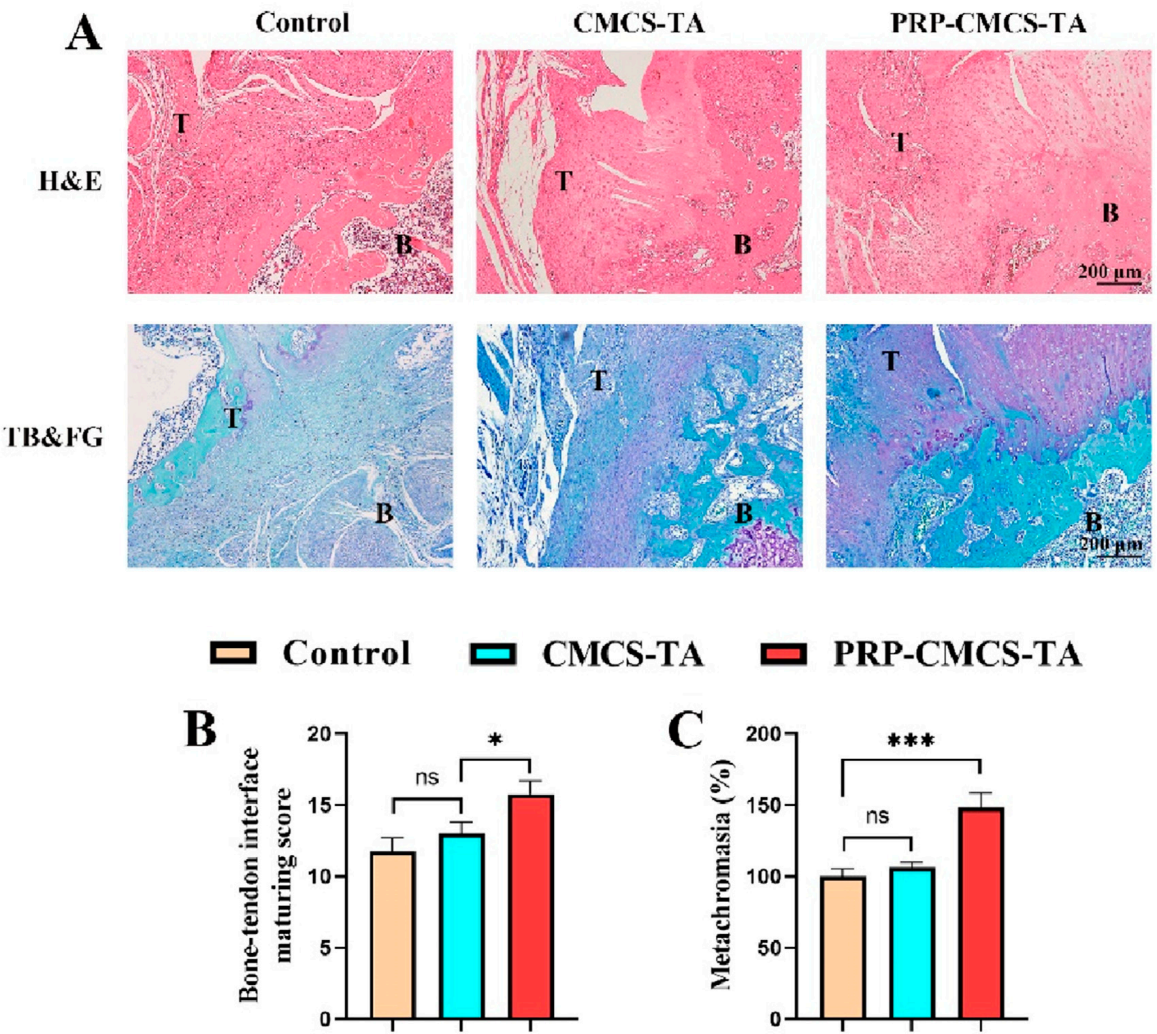
**(A)** Morphological analysis of newly formed tendon - bone interface tissue after 8 weeks of treatment, including H&E staining and TB&FG staining. T: Tendon, B: Bone. **(B)** Bone-tendon interface maturing score of different treatment groups. **(C)** The area of newly formed fibrocartilage in the different treatment groups. Data are presented as mean ± SD (n = 4 independent animals per group). Statistical analysis was performed using one-way ANOVA. *p < 0.05, ***p < 0.001*,*”ns” represents no significant difference.

Figure 9 presents the morphological analysis of newly formed tendon - bone interface tissue after 8 weeks of treatment, using H&E and TB&FG staining. In H&E staining, the control group shows disorganized tissue with poor integration between tendon and bone. The CMCS - TA group has improved tissue structure but still has a relatively wide gap between tendon and bone. The PRP-CMCS-TA group demonstrates well-organized tissue with a narrower gap and better integration between tendon and bone. In TB&FG staining, the control group has limited collagen fibers and mineralized tissue. The CMCS - TA group shows more collagen fibers but less mineralized tissue. The PRP-CMCS-TA group has abundant collagen fibers and more mineralized tissue, indicating advanced tissue regeneration. The semi-quantitative histological scores for the healing of the tendon-bone interface are presented in Figure 9B. Statistical analysis demonstrated that the PRP-CMCS-TA group achieved a significantly higher total score compared to the CMCS-TA group (p < 0.05). Furthermore, quantitative analysis of the metachromatic area in TB-stained sections revealed a significantly larger stained area in the PRP-CMCS-TA group compared to the Control group (p < 0.001). (Figure 9C). These results collectively demonstrate that the PRP-CMCS-TA hydrogel significantly enhances the quality of tendon-bone interface regeneration, promoting the formation of a more native-like transitional tissue. Overall, the PRP-CMCS-TA hydrogel effectively promotes tendon - bone interface healing, enhancing tissue organization and mineralization.

## 4 Conclusion

In this study, we successfully developed and evaluated a novel PRP-CMCS-TA composite hydrogel for the repair of rotator cuff tendon-bone interface (TBI) injuries. The findings demonstrate that this composite hydrogel not only exhibits excellent biocompatibility and sustained release of bioactive factors but also significantly promotes both osteogenic and tendogenic differentiation *in vitro*. In a rat model of rotator cuff repair, the PRP-CMCS-TA hydrogel markedly enhanced bone regeneration, improved biomechanical strength, and facilitated the formation of a more organized and continuous tendon-bone interface, as confirmed through comprehensive micro-CT, biomechanical, and histological analyses.

Our results align with and extend previous efforts in the field of biomaterial-based strategies for TBI regeneration. For instance, studies utilizing PRP alone have shown limited efficacy due to rapid clearance and lack of mechanical support (Khandan-Nasab et al., 2025). Similarly, hydrogels based on chitosan or its derivatives have demonstrated promising biocompatibility but often lack the necessary bioactivity to drive robust integrative repair. The innovation of our study lies in the synergistic combination of a CMCS-TA hydrogel network with PRP. This design effectively addresses key limitations of PRP delivery by providing a sustained release platform, while the TA component enhances structural stability and potentially stabilizes growth factors, a challenge noted in other systems (Niu et al., 2024). The resulting functional outcomes in terms of bone formation (BV/TV, BMD) and biomechanical strength appear superior to those reported in studies using single-component systems, underscoring the advantage of a combined bioactive and bioadhesive approach.

A key strength of this work is the multifaceted evaluation approach, encompassing *in vitro* cytocompatibility, bioactivity, and controlled release assessments, validated by robust *in vivo* functional and morphological outcomes in a established animal model. The use of a CMCS-TA system for PRP delivery presents a novel and translatable strategy.

However, this study has several limitations. First, the animal model involved healthy, young rats, which may not fully replicate the compromised healing environment (e.g., poor vascularity, chronicity) often seen in clinical patients with rotator cuff tears. Second, the long-term fate and degradation kinetics of the hydrogel *in vivo* were not evaluated beyond the 8-week time point, which is important for understanding its complete translational profile. Third, while the sustained release of key growth factors was demonstrated, the specific molecular mechanisms through which the PRP-CMCS-TA hydrogel orchestrates cellular processes and promotes interface regeneration warrant further investigation.

In summary, the PRP-CMCS-TA hydrogel represents a highly promising and multifaceted strategy for augmenting rotator cuff repair. By addressing critical challenges of biological integration and sustained growth factor delivery, this study provides a solid foundation for the future development of effective bioactive scaffolds for soft tissue-to-bone healing.

## Data Availability

The raw data supporting the conclusions of this article will be made available by the authors, without undue reservation.
